# The need for educational intervention for breastfeeding women and the professional practice of midwives in France to promote breastfeeding: A joint explanatory study

**DOI:** 10.18332/ejm/191176

**Published:** 2024-12-11

**Authors:** Mehrnoosh Yazdanbakhsh, Vincent De Andrade, Laurence Spiesser-Robelet, Rémi Gagnayre

**Affiliations:** 1 Educations and Health Promotion Laboratory, Sorbonne Paris Nord University, Villetaneuse, France

**Keywords:** learning environment, breastfeeding skills, teaching skills, promotion of breastfeeding, education strategies, perception of usefulness

## Abstract

In France, breastfeeding prevalence is high at birth, but its continuation remains low compared with other high-income countries, despite worldwide public health recommendations. Midwives offer parenting classes in an experimental manner without considering the importance of education in their interventions. The objectives of this study were to identify the teaching strategies and learning environments offered by midwives and their effect on women's perception of usefulness and their breastfeeding competence, to assess midwives' perception of usefulness and their pedagogical competencies. A comparative mixed study of 20 hospital midwives and 53 breastfeeding women (at 3 and 30 days postpartum) was conducted between January and August 2022 in two maternity units in France. Comparing the two periods, positive effects were found about breastfeeding women’s level of knowledge: usefulness of learning theoretical (p=0.01) and practical (p=0.00) knowledge; and their breastfeeding management: signs of lactation (p=0.00), breast engorgement (p=0.04), and behavior (p=0.04). It positively reinforced the development of self-esteem (p=0.00) and commitment to breastfeeding (p=0.00). Midwives expressed strong motivation to use an appropriate teaching strategy and provide a supportive learning environment for women to improve their educational interventions (mean motivation score 7.7/10). The study results can promote research to examine educational interventions modeled according to the theories in education. Critical realism can be used to evaluate these interventions to elucidate how a program based on educational engineering can contribute to breastfeeding promotion and achieving the 2030 goals of WHO.

**CLINICAL TRIAL REGISTRATION:**

The study was registered on the official website of ClinicalTrials.gov

**IDENTIFIER:**

ID NCT05271812

## INTRODUCTION

The scientific community widely acknowledges the benefits of exclusive breastfeeding (BF) on infants and their mothers. Breast milk protects the newborn against gastrointestinal and otolaryngological infections, food allergies, atopic dermatitis, and asthma^1,2^. The Omega 3 and 6 type polyunsaturated fatty acids in breast milk promote the cerebral^3,4^ and neurocognitive development of the newborn^5^. Long-term exclusive BF has been reported to act as a protective factor against unexpected deaths in newborns^6^.

The benefits of BF for mothers include uterine involution and faster postpartum weight loss. Studies have reported that BF acts as a protective factor against the occurrence of breast and ovarian cancer, cardiovascular diseases, and type 2 diabetes^7^.

The promotion of BF has become a global public health target. The World Health Organization (WHO) aims to ensure that 50% of newborns benefit from exclusive BF from birth by 2030^8^. Since 2018, WHO has also recommended that programs and/or education interventions to promote BF be evaluated^9^.

In France, despite the increase in the exclusive BF rate at birth, from 54.6% in 2016^10^ to 56.3% in 2021, the exclusive BF rate continues to decrease to 38.4% at two months after birth^11^. This progress remains inadequate compared with that reported in countries where the exclusive BF rate at birth and two months after birth is >90%^12^.

Although many health plans^13,14^ implemented by the French government recommend BF to address nutritional health inequalities, these plans continue to be comprehensive and do not propose a program, much less an indication of the strategies for implementing promotional actions.

Currently, aside from health checkups specific to BF conducted by lactation consultants, the only BF promotional action provided for in an educational program is called ‘Preparation for Birth and Parenthood (PBP)’, proposed and implemented by midwives^15^. This program comprises eight information and education sessions, each approximately 1 hour long, one specific for BF. These sessions are not mandatory and offered to antenatal couples only. Midwives were free to choose the themes to be addressed, the information to be transmitted, the teaching strategies to be applied, and the learning environments to be created. There is heterogeneity and disparity in midwifery educational interventions, especially since no reference to conceptual frameworks of educational engineering enabled midwives to better understand the complexity of the promotional and educational dimensions.

The objective of the present study is to identify the teaching strategies and learning environments offered by midwives, evaluate their teaching skills, evaluate and explain the perception of the usefulness of these educational actions with breastfeeding women, and evaluate and explain the breastfeeding women’s self-efficacy and self-esteem in managing their breastfeeding and their commitment to breastfeeding.

## METHODS

### Study design

This mixed concomitant and explanatory study^16^ includes quantitative and qualitative analytical approaches. The quantitative section assesses the usefulness of midwifery interventions through the level of understanding, importance, and satisfaction of breastfeeding women who received these interventions. The level of women’s self-efficacy and self-esteem in managing their breastfeeding in relation to the educational intervention and the personal commitment intention to breastfeed was also assessed. All criteria were evaluated using 4-point Likert scales and 10-point numerical scales. In the qualitative section, the verbal explanations given by BF women and midwives are categorized into themes and sub-themes as they justify their answers during the interviews.

### Study population

The participant sample included women who gave birth in two maternity wards of the University Hospital in Normandy and Ile de France and midwives practicing there. The choice of both maternities in different health districts enabled us to favor the diversity of the populations in the study. The two Maternity Centers have identical operational processes and care organizations; however, their districts are very different.

A declaration of the research methodology has been made to the National Commission for Computing and Liberties (CNIL) and Sorbonne University Research Ethics Committee, the registration numbers are respectively 2224293 and CER-2021 -119. Also, this search is registered at https://clinicaltrials.gov/: NCT05271812.

### Participant selection and recruitment procedure

*Selection and recruitment for BF women*

The decision to interview women who had given birth and were BF, who participated in the educational intervention on BF was justified to assess the effects of those educational interventions on BF promotion on the newborn’s 3rd day and one month after birth. BF continuation at one month after the birth of the newborn permitted us to analyze the usefulness of educational interventions according to the perception of BF women and their feeling of competence to manage BF.

To avoid the influence of obstetrical pathologies on BF choice and maintenance and its subsequent conduct, we conducted the survey among French-speaking adult women with low obstetrical risk, BF and/or previous BF experience and who benefited from the educational session on BF. Even though the presence of co-parents is strongly recommended, it is not mandatory during educational interventions. Therefore, we did not include co-parents in this study.

Eligible BF women were informed by providing the necessary information, and a non-objection letter was obtained from them on the 1st day of their delivery in the postpartum services. A 24-hour reflection period was respected so that the women could decide to participate in the study. After collecting the non-objection, the women answered the same questions administered by the project investigator during a face-to-face meeting on the 3rd day and the 1st month after the birth of their newborns by telephone during appointments organized by the project investigator.

*Selection and recruitment for midwives*

The midwives were informed before the start of the survey based on their presence in the postpartum services. Eligible midwives were informed through oral communication. After obtaining their consent, the midwives answered a questionnaire administered by the project investigator during regular visits to the postpartum services. A 24-hour reflection period was also granted to the midwives to confirm their participation in the study. Interviewing hospital midwives is justified because the couples’ education in parenthood is an integral part of French hospital missions. It also seemed relevant to interview only hospital midwives who had experience facilitating educational interventions in BF during the antenatal period to identify the strategies they use in their educational intervention, evaluate their perception of the usefulness of their educational interventions, and appreciate their pedagogical skills to promote BF.

### Study measurement tools and data collection

For both BF women and hospital midwives, data collection was performed using a questionnaire designed for the needs of this study. BF women’s usefulness perception vis-à-vis the educational interventions was quantitatively and qualitatively measured at day 3 (D3) and day 30 (D30) postpartum.

*BF women measurement and data collection*

The questionnaire was divided into five sections: 1) To identify teaching strategies and the learning environment, which were assessed using nine items on a four-point Likert scale; 2) The item’s usefulness was evaluated using four items on a four-point Likert scale^17^ within which an open-ended question is posed to allow women to justify their choice. The feeling of competence is defined as the person’s ability to mobilize their intrinsic and extrinsic resources in a family of situations to obtain the most relevant result^18^; 3) We assessed the feeling of self-efficacy or personal efficacy^19^. This was measured based on the BF Self-Efficacy Scale-Short Form (BSES-SF) proposed by Dennis et al.^20^ in 2003. The French version of the BSES-SF was validated by an expert committee (translators, health professionals, lactation consultants, and researchers)^20^. A skill’s emotional component involves measuring self-esteem with respect to one’s learning. It gradually builds through others’ positive and negative evaluations or personal self-evaluation by comparing the targeted goals with the results^21^. The self-efficacy construct is evaluated by administering nine items^20,21^ on a four-point Likert scale. An open-ended question is posed to permit women to justify their selection; 4) The self-esteem assessment employs a 10-point scale containing six items^19,20^. An open-ended question is posed to enable women to justify their responses; and 5) The personal commitment intention to breastfeed is evaluated through the administration of three items^22^ on a 10-point numeral scale. An open-ended question is posed to permit women to justify their selection.

*Midwives’ measurement and data collection*

The questionnaire was structured to identify teaching strategies and learning environments. The same criteria (nine items) are suggested for midwives. Each item is rated on a 10-point numerical scale according to its importance. An open-ended question is posed to allow midwives to justify their choice of teaching strategies and learning environment.

### Data analysis

*Quantitative analysis technique*

The quantitative variables were described by frequencies and percentages using Excel software. The comparison between the quantitative values was provided by uncorrected chi-squared tests (χ^2^). When an expected value was ≤5, the Fischer’s exact test was used using OpenEpi software. The result was considered significant when the confidence interval was 95%, and the bilateral p-value was <0.05.

*Qualitative analysis technique*

According to Paillé and Muchielli^23^, it is based on the thematic analysis. It is a coding and categorization of verbatim responses collected through open questions. The first analysis was performed manually using a developed analysis grid. To ensure the accuracy of the units of meaning and their connections categorization, two researchers from LEPS UR 3412 performed the thematic coding double reading, experts in BF and qualitative analysis issues. Finally, in case of disagreement with the project investigator, a third senior researcher is appointed to check the accuracy of the analysis of the verbatim comments regarding the study’s goals and the evaluation criteria. The presentations of the figures are drawn with the Xmind software free version.

### Quantitative data collection

*The general profile of the study population*

We included 20 hospital midwives and 53 BF women. Tables 1 and 2 present the general profile of the study population. Only 25 (47%) women had experience of exclusive or mixed BF. The average duration of BF, all types combined, is 3 months and 15 days. The duration of BF varies between 3 weeks and 24 months. Most interviewed women (n=52/53; 98%) decided to breastfeed before the newborn’s birth. The most common reasons for this decision were benefits for the newborn (n=50/53; 94%), professional advice (n=21/53; 46%), including during PNP (postnatal prophylaxis) sessions (n=13/53; 24%) and their self-training (n=27/53; 50%).

**Table 1:** The general characteristics of breastfeeding women in the Normandy and Ils de France regions, 2022 (N=53)

*Characteristics*	*Categories*	*n*	*%*
**Age** (years)	18–20	0	0
	21–30	16	30.19
	31–40	33	62.26
	41–45	3	5.66
	>45	1	1.89
**Education level**	Primary school	0	0
	A levels	10	18.87
	Diploma of Higher Education	6	11.32
	BA, BS/BSc	10	18.87
	MA/MS/MSc (NVQ Level 5)	20	37.73
	PhD	7	13.21
**Socio-professional categories according to INSSE**	Farmer-operator	2	3.77
	Craftsman-merchant	4	7.55
	Executive-intellectual profession	23	43.39
	Intermediate occupation	4	7.55
	Employee	19	35.85
	Worker	1	1.89
**Professional position during the current pregnancy**	Yes	51	96.23
	No	2	3.77
**Parental leave, following the current pregnancy**	Yes	14	26.42
	No	39	73.58
**Family/couple status**	Yes	53	100.00
**Number of dependent children**	0	27	50.94
	1	15	28.30
	2	11	20.76
	>2	0	0
**Breastfeeding experience** (postpartum)	Yes	25	47.17
	No	28	52.83
**Type of AM**	EBF	18	33.96
	AMM	7	13.21
	>6 months	14	56.60

EBF: exclusive breastfeeding. AM: … AMM: … INSSE: …

**Table 2:** Characteristics of hospital midwives in Normandy and Ils de France regions, 2022 (N=20)

*Characteristics*	*Categories*	*n*	*%*
**Age** (years)	23–30	3	15
	31–40	5	25
	41–45	4	20
	>45	8	40
**Professional experience** (years)	<1	0	0
	1–5	1	5
	6–10	5	25
	11–15	2	10
	16–20	5	25
	21–25	0	0
	26–30	3	15
	>30	4	20
**Specific training on breastfeeding**	IBCLC	0	0
	University diploma in breastfeeding	1	5
	Breastfeeding MOOC	1	5
	Institutional training	11	55
	No training	7	35
**Health education experience**	PNP sessions	9	45
	Information meeting on BF	4	20
	Only during initial training	7	35

IBCLC: … MOOC: … PNP: …

Table 2 shows the general profile of the midwives interviewed. Forty percent of midwives were aged >40 years, 35% (n=7/20) had professional experience of >26 years, and 45% (n=9/20) had facilitated educational interventions.

Table 3 presents the pedagogical characteristics of educational interventions according to breastfeeding women and midwives identified after the interviews.

**Table 3:** Pedagogical characteristics of educational interventions according to breastfeeding women and midwives in Normandy and Ils de France regions, 2022

*Evaluation criteria*	*Categories*	*Breastfeeding women (N=53)*		*Midwives (N=20)*	
		*n*	*%*	*n*	*%*
**Organizational of the session**	Small group <10 with the presence of a co-parent	15	28.30	16	70
	Individual without the presence of a coparent	12	22.64		
	Individual with the presence of co-parent	10	18.86	4	30
**Training location**	Private midwife Cabinet	38	71.70		
	Equipped rooms in maternity wards	15	28.30	16	80
**Session period**	7 months pregnant	19	35.85		
	8 months pregnant	11	20.75		
	Antenatal and postnatal			16	80
**Educational goals** (theoretical contributions)	Benefit for the newborn	42	79.26	19	95
	Complications: cracks	39	73.58	18	90
	Complication: breast engorgement	32	60.38	13	65
	Lactation physiology	29	54.23	19	95
	Benefit for the mother	20	37.73	17	85
**Educational goals** (practical workshop contributions)	Demonstration, without women practice	14	26.41	5	25
	Demonstration, with women practice	14	26.41	13	65
	Demonstration, with women practicing and educational video projection	4	7.55		
**Teaching methods**	Active teaching under the form of discussion throughout the session	32	60.28	14	70
	Passive teaching under the form of lectures with discussion time at the end of the session	10	18.89	6	30
**Teaching tools**	Breastfeeding booklet	24	45.28	11	55
	Flyers/photos	9	16.98		
	Educational film/video			14	70
**Duration of the session** (min)	45			6	30
	60	27	50.97	10	50
	>60	11	27.76	3	15

*Assessment of breastfeeding women*

Usefulness perception, self-efficacy, and personal commitment intention to breastfeed vis-à-vis educational interventions were sought from BF women on the 3rd and 30th day after the birth of their newborns. The mean, percentage, and standard deviation are calculated for each period. For performing the comparison tests (χ^2^ or Fischer’s exact test), we divided the values assigned on the Likert scale into two categories: 1–2 = minimum value and 3–4 = maximum value. We also divided the values assigned on the 10-point numerical scale into three categories: 1–3 = minimum value, 3–7 = average value, and 7–10 = maximum value. The χ^2^ comparison test permitted the calculation of the p-values. All the quantitative breastfeeding women levels results are presented in Table 4.

**Table 4:** Assessment levels of breastfeeding women’s perceptions of usefulness and skills-feeling between day 3 and day 30 postpartum regarding educational intervention in Normandy and Ils de France regions, 2022 (N=53)

*Evaluation criteria*			*Scores^§^*	*D3 n*	*D30 n*	p* (D3-D30)
**Breastfeeding women usefulness perception**	Adaptability/educational needs	Theoretical contributions	1–2	11	14	**0.02**
			3–4	42	18	
		Practicing gestures (practical workshop)	1–2	20	19	0.05
			3–4	33	13	
	Importance granted/educational needs	Theoretical contributions	1–2	12	3	0.12
			3–4	41	29	
		Practicing gestures (practical workshop)	1–2	7	4	0.92
			3–4	46	28	
	Motivation generated/educational needs	Theoretical contributions	1–3	10	6	0.62
			4–7	23	17	
			8–10	20	9	
		Practicing gestures (practical workshop)	1–3	10	5	**0.01**
			4–7	32	11	
			8–10	11	16	
	General satisfaction	Theoretical contributions	1–2	8	9	0.14
			3–4	45	23	
		Practicing gestures (practical workshop)	1–2	19	7	0.41
			3–4	49	25	
**Breastfeeding women skillsfeeling**	Self-efficacy feeling	Explain the lactation physiology	1–2	22	3	**0.00**
			3–4	31	29	
		Know the signs of effective breastfeeding	1–2	4	0	0.11
			3–4	49	32	
		Act as a consequence	1–2	5	0	0.07
			3–4	48	32	
		Practice good breastfeeding gestures	1–2	7	0	**0.03**
			3–4	46	32	
		Act as a consequence	1–2	5	0	0.07
			3–4	48	32	
		Know the signs of cracks	1–2	16	0	**0.00**
			3–4	34	32	
		Act as a consequence	1–2	9	0	**0.01**
			3–4	44	32	
		Know the signs of breast engorgement	1–2	23	2	**0.00**
			3–4	30	30	
		Act as a consequence	1–2	21	2	**0.00**
			3–4	32	30	
	Self-esteem feeling	Acknowledge to have qualities	1–3	0	0	**0.00**
			4–7	35	8	
			8–10	18	24	
		Succeeding in breastfeeding/difficult task	1–3	4	0	0.41
			4–7	20	9	
			8–10	29	23	
		Breastfeed comfortably/surroundings	1–3	8	1	0.07
			4–7	14	5	
			8–10	31	26	
		To feel well considered/surroundings	1–3	5	0	**0.00**
			4–7	16	1	
			8–10	32	31	
		Feeling proud/breastfeeding	1–3	3	0	**0.00**
			4–7	32	4	
			8–10	18	28	
		Acknowledge facing time	1–3	0	0	0.05
			4–7	9	1	
			8–10	44	31	
	Personal commitment intention	Feeling comfortable with breastfeeding	1–3	15	0	**0.00**
			4–7	27	1	
			8–10	11	31	
		Be satisfied with the management	1–3	2	1	0.05
			4–7	19	4	
			8–10	32	27	
		Wanting to continue breastfeeding	1–3	0	1	0.40
			4–7	1	1	
			8–10	52	30	
			3–4	49	25	

§ Usefulness scores: 1 = inappropriate, 2 = not very appropriate, 3 = appropriate, 4 = very appropriate. Assigned value according to nuance points: 1–3 = minimum value, 3–7 = average value, 7–10 = maximum value.

* Bilateral uncorrected chi-squared. Statistical significance at p<0.05.

*Assessment of midwives*

Midwives were interviewed regarding the teaching strategies they use to implement their educational interventions. They had to define the perception of usefulness they attribute to the teaching strategies they implement, and they had to evaluate their pedagogical skills to promote BF. Table 5 shows the usefulness perception vis-a-vis their teaching strategies according to the degree of importance and the motivation score for the intervention’s development.

**Table 5:** Assessment levels of midwives’ perception of usefulness for teaching strategies, learning environment, and motivation, in Normandy and Ils de France regions, 2022 (N=20)

*Criteria evaluated*	Pedagogical competence assessment levels*	*n*	*%*	*Motivation on a 10-point scale Score (range)*
Applied teaching methods to provide theoretical knowledge	1–2	0	0	7.90 (7–10)
	3–4	20	100	
Educational goals	1–2	1	5	7.75 (1–10)
	3–4	19	95	
Learning environment	1–2	1	5	7.30 (1–10)
	3–4	19	95	
Educational tools and supports	1–2	1	5	8.15 (4–10)
	3–4	19	95	
Applied educational methods for the demonstration of breastfeeding gestures	1–2	2	10	7.95 (5–10)
	3–4	18	90	
Teaching organization methods	1–2	3	15	7.20 (5–10)
	3–4	17	85	

* Usefulness scores: 1 = inappropriate, 2 = not very appropriate, 3 = appropriate, 4 = very appropriate.

### Qualitative data collection

*Verbatim thematic analysis of breastfeeding women*

The open-ended questions at the end of each quantitative evaluation allowed women to explain their perceptions of the usefulness of the educational interventions and their impact on their feelings of self-efficacy, self-esteem, and commitment to continue breastfeeding. The thematic categorization of BF women’s verbatim comments revealed that educational interventions influenced their perception of usefulness and their skills in BF in three ways.

The intervention’s positive influence was mentioned by all interviewed women for the contribution of knowledge (n=53/53; 100%, verbalized 397 times) and acquisition of BF management (n=45/53; 84%). The educational intervention exerted a positive influence on women’s psycho-affective state (n=40/53; 75%, verbalized 203 times). They mentioned the influence of the intervention on their motivation building, self-efficacy feeling, self-esteem, and personal commitment to BF. Other themes were identified in connection with a favorable influence of educational interventions such as increased motivation (n=31/53; 58%, verbalized 115 times), positive perception of BF (n=21/53; 39%, verbalized 68 times), decision-making to breastfeed (n=18/53; 33%, verbalized 63 times), assimilation and understanding of the lactation physiology (n=17/53; 32%, verbalized 60 times) and the presence of a co-parent during the intervention (n=10/53; 18.86%, verbalized 10 times).

Despite a favorable perception vis-à-vis the educational interventions in general, for some women, the content and the way the midwives transmitted this information created a rather negative perception (n=8/53; 15%, verbalized 127 times). A negative perception was evoked as well among (n=13/53; 24%) vis-a-vis the latter.

**Figure 1:**
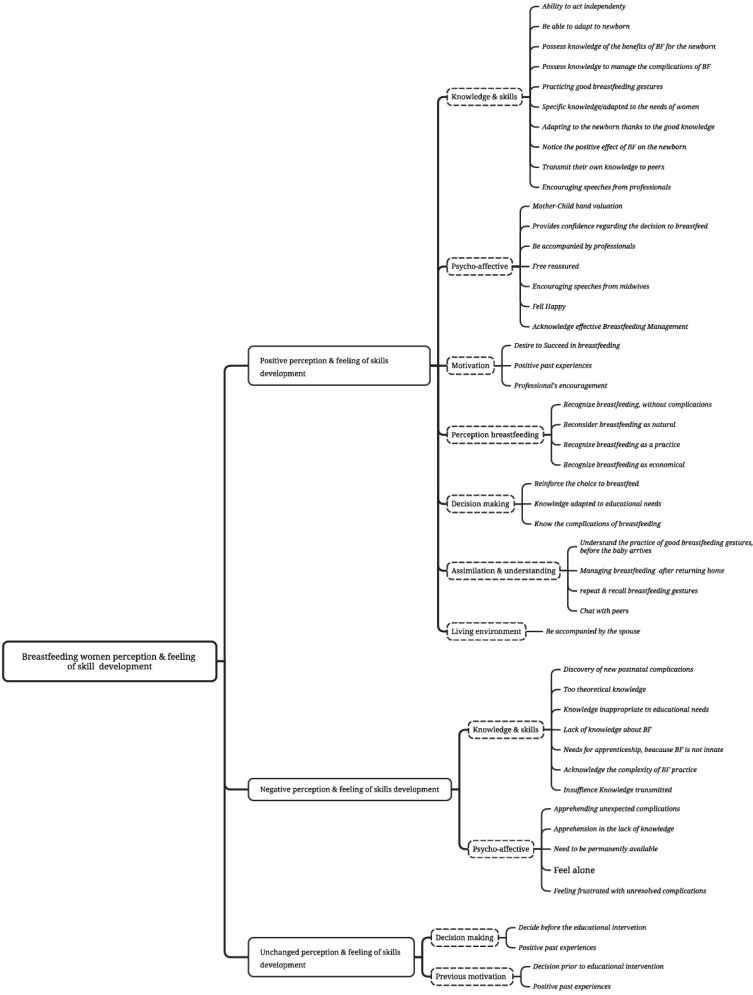
Verbatim thematic analysis of the breastfeeding women’s comments

Finally, the positive or negative perception of women regarding educational interventions and their influence was not found when women decided their choice before the educational sessions or had a positive experience of BF (n=19/53; 35%); no positive or negative declaration was noted regarding the usefulness perception and the skills acquisition feeling. Figure 1 presents the thematic analysis of the BF women verbatim comments.

*Verbatim thematic analysis of midwives*

The open-ended questions at the end of each quantitative evaluation allowed midwives to explain their perceptions of usefulness perception and their pedagogical skills. The midwives’ verbatim comments thematic categorization highlighted three main headings (motivation, pedagogical posture, and professional positioning), thus explaining their usefulness perceptions and pedagogical skill feelings about their educational interventions.

Motivation is the 1st heading identified (n=20/20; 100%, verbalized 90 times). The midwives mentioned it through themes such as transmitting knowledge (n=20/20; 100%, verbalized 46 times), building an environment favorable to learning (n=17/20; 85 %, verbalized 35 times), interacting with women (n=12/20; 22%, verbalized 20 times), using interactive educational tools (n=9/20; 45%, verbalized 10 times) and performing the demonstration of BF gestures and positions (n=7/20; 35%, verbalized 15 times).

The interviewed midwives explained the educational interventions’ usefulness perception and the feeling of pedagogical skill when they mentioned their choice of educational strategy. This 2nd heading seemed important when they expressed the usefulness of their interventions and pedagogical skill. For 85% (n=17/20) of them, their pedagogical position seems to be close to the learning paradigm, while for 75% (n=15/20) of them, their pedagogical position is rather inspired by the teaching paradigm.

**Figure 2:**
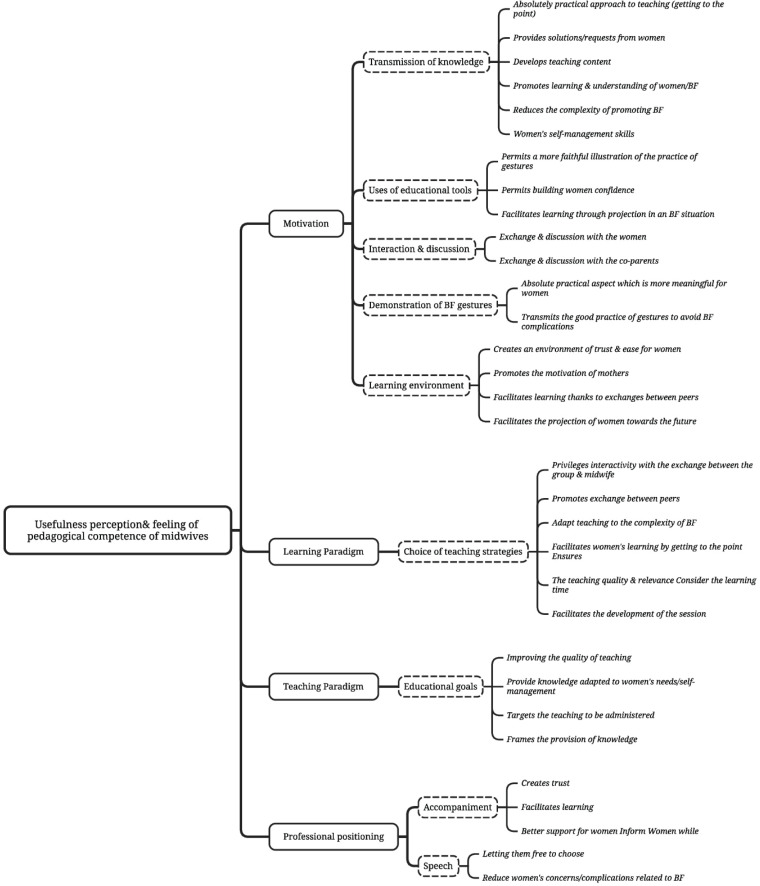
Verbatim thematic analysis of midwives’ comments

Professional positioning is the third item identified by the interviewed midwives in connection with the usefulness perception of their educational intervention and their teaching skills (n=12/20; 50%, verbalized 25 times). Related to this topic, other themes emerged, including supporting women in their choice (n=7/20; 35%) and the quality of speech during the intervention (n=5/20; 25%). Figure 2 presents the thematic analysis of the midwives’ verbatim comments.

## DISCUSSION

### Teaching strategies and learning environment

Many women and midwives have noted and explained the use of active teaching methods. The organization of educational sessions, in the form of discussion and exchange in small groups of less than ten people, with the presence of co-parents, declared by a minority of women and mostly midwives, also confirms the midwives’ pedagogical position, centered on women learning through exchanges, the source of socio-cognitive conflicts^24^. The pedagogical strategies chosen refer more to constructivism.

When midwives attempt to learn the positions and BF gestures, the adopted position is inspired by the information transmission paradigm. For them, the teacher’s role is a one-way transmission of ‘know-how’. Midwives maintain a central place in the educational strategy. The midwives mentioned the demonstration of gestures during practice time for women. However, women’s feedback on the methods proposed for this apprenticeship is very diverse. Only a minority of women explained that they practice BF gestures with a doll. The same proportion of women explained they had observed only a simple demonstration of gestures. Finally, some interviewed women declared that they practiced gestures and observed educational videos in the midwife’s presence.

These findings confirm the results of a literature review^25^ that highlighted this diversity of pedagogical strategies centered on teaching or learning with differences in reference to pedagogical trends.

Though the use of written teaching aids and tools (booklet, leaflet) and/or visual and interactive (photos, educational videos, and testimonial films) was addressed by over half of midwives, only a part of the women could benefit from written support. This result raises issues about the midwives’ recognition of the pedagogical interest of materials that facilitate learning. Indeed, the combination of several categories of tools and support was identified in the same review of scientific studies as one of the factors promoting learning^25^. Notably, written materials (booklet, brochure, poster), material for BF positions understanding doll (baby, childcare material), visual materials (color or black and white image, film and educational video)^26^ and finally, interactive and playful supports^27^ (computerized platform, web site, telephone application, text message-SMS) were used in the majority of the educational interventions thus influencing BF duration in the short- and medium-term.

The duration and the rhythm of learning have been reported for their pedagogical interests in the repetition of information and the transmission of knowledge by midwives. Indeed, over half of the midwives interviewed acknowledged that successful education of women in BF would require learning time at a steady pace to ensure antenatal and post-education continuity. However, BF women reported they have benefited from only one educational session about antenatal BF. This finding undoubtedly raises questions about the management of postnatal BF women. Especially since the repeated and close educational interventions right after leaving the maternity ward postpartum in the form of telephone calls or close home visits positively influenced the duration of BF in the medium-term^28^.

The midwives mentioned the content of the knowledge to be transmitted and the interest in its definition thanks to the educational objectives. Although they mentioned the prioritization of certain themes, such as the benefits for the newborn and the mother, the physiology of lactation, and the cracks that occurred. Only less than half of women reported they received information about lactation and the benefits for the mother. The same is true for very frequent complications such as breast engorgement, which were not addressed in only half of the women. This discrepancy in the results between the midwives’ statements and the women’s observations about the content of the interventions underlines a generalization of the information transmitted. However, studies^29,30^ have shown that when women have a good level of knowledge specific to BF, like lactation physiology and the most frequent complications such as breast pain during BF, cracks and/or breast engorgement were for longer term BF.

### Women’s usefulness perception and BF skills development

BF women’s usefulness perception vis-à-vis the educational interventions was quantitatively and qualitatively measured at D3 and D30. The women perceived the usefulness of the interventions for their level of adaptability to their educational needs. They granted the usefulness of these interventions according to their positive influence on their motivations to practice BF gestures before the newborn’s arrival. However, they gave the same level of importance to the accessibility and understanding of the intervention content, the practice of the gestures, and the influence of the theoretical contributions on their motivation to breastfeed. The verbatim thematic analysis confirmed these findings since the usefulness of the interventions, both theoretically and practically, was perceived as a lever to facilitate the choice of BF and the development of BF skills for better remote management.

BF women were interviewed about their feelings about BF skills at D3 and D30. A global feeling of self-efficacy was identified among women from the 3rd day postpartum. A significant difference in values was found between D3 and D30 for lactation physiology, knowledge of signs of effective sucking, cracks, and breast engorgement. The highest scores were assigned at D30. These results would confirm the development of a self-efficacy feeling rather at one one-month distance. This result corroborates the selection of knowledge transmitted by midwives during antenatal educational sessions. In other words, the physiology of BF and its complications are very little discussed during antenatal educational sessions. Moreover, women’s education time by professionals in the early postnatal period before leaving the maternity ward is reduced. Hospitalization times are drastically reduced due to early discharge in low-risk women (stay of 48 hours after childbirth). The average length of stay in French maternity wards continued to decrease between 2016 and 2021^11,12^. Consequently, the follow-up of women leaving the maternity ward is centered more on postpartum care and monitoring.

Even though the comparison of values was insignificant for ‘feeling successful in her BF’, ‘being comfortable with the installation’, and ‘coping with the time’, the educational interventions globally favored self-esteem development from D3 to ‘acknowledge having qualities’, and ‘feel well considered and be proud’. Educational interventions have a positive effect on self-esteem development. The verbatim thematic analysis highlighted the same result since positive feeling was the 2nd theme identified by BF women.

Personal commitment intention in longer term BF, in connection with educational sessions received, was substantially similar between the 3rd and 30th day ‘to be satisfied with the management’, and ‘to want to breastfeed’. They felt ‘comfortable with their BF’. The thematic analysis revealed the positive influence of the sessions on this item since motivation, assimilation, and understanding were themes explained by women to justify their commitment to BF.

### Midwives’ usefulness perception and pedagogical skills development

Midwives’ usefulness perception vis-à-vis their teaching strategies and the learning environment is considered an important, even very important, usefulness. This perception is passed on to the teaching strategies concerning the contribution of theoretical knowledge and the demonstration of BF gestures, as well as the objectives and the teaching tools. They expressed their strong motivation to apply an appropriate teaching strategy and provide an environment favorable to learning for women to develop their interventions. Although these findings testify the midwife’s awareness of the educational value of their interventions, the verbatim thematic analysis rather raises a position on a classic transmission in terms of BF. Midwives feel the usefulness of their interventions in transmitting exact knowledge (1st identified theme) and as a lever to establish a relationship of care and trust (2nd and 3rd themes mentioned). The usefulness of their interventions was also perceived as a preventive approach to avoid BF complications. Here, the biomedical posture largely takes precedence over an educational posture, which is very strongly explained by the midwives.

In general, midwives assess their skills according to their BF expertise and professional motivations. Though other subthemes like ‘speech’ and ‘coaching’ were expressed in relation to their teaching skills, they claim more ‘caregiver’ than ‘educator’ role with women. In this sense, all the midwives interviewed had already facilitated (alone or accompanied) these educational sessions, but none of them had been trained in pedagogy. According to our literature review, the skills of trainers in pedagogy have shown a positive impact on the duration of exclusive BF in the short- and medium-term.

### Converging themes between women’s educational needs and the teaching strategies proposed by midwives

At the end of these analyses, four shared themes are identified. Both the women and the midwives interviewed seem to favor building BF knowledge. Despite the midwives’ reluctance in not wanting to transmit selective information so they would not ‘generate a feeling of concern among women’, the need for BF knowledge is the 1st theme quoted by women. The contribution of theoretical knowledge on the benefits of BF for the mother and her newborn, lactation physiology, and the most frequent complications are essential not only to promote the choice of BF and to support women’s confidence in their decision, but also this contribution influences the development of self-efficacy, self-esteem, even women personal commitment in their BF.

The second shared point of view would be learning BF gestures through practice. The exact learning of BF gestures through practice before the arrival of the newborn seems to be essential to provide a sense of assurance to pregnant women. Similarly, the studies^30,31^ selected in the review of the literature highlighted that the practice workshops with women exercising, combined with the projection of educational videos or films, would better permit assimilation and understanding of BF gestures.

Interactivity and permanent access to information would be the 3rd point of view shared. The introduction of new information and communication technology made teaching and pedagogy evolve. These technologies have demonstrated their performance in the patients’ therapeutic education. Though not all the women spoke about this subject, half of them visited specific BF websites for information and training. Continuous access to accurate information is important for improving women’s skills development. In this sense, studies^26^ highlighted that permanent access to knowledge during early pregnancy promotes BF duration in the short- and medium-term. Providing women with a free access platform specialized^32-34^ in BF could facilitate learning and compensate for the lack of educational support for postnatal BF women. The latter significantly influenced BF’s short- and medium-term duration.

Educational support throughout BF would be the last point of view shared by the women and midwives interviewed. The need to manage postnatal BF frequent complications, as well as the need to know about lactation physiology, was systematically mentioned by women; the midwives also mention these points of view. BF women and midwives acknowledge the positive influence of educational support during the course of BF’s duration. In fact, the results of the literature review^25^ similarly show that the BF education programs offered by the community-hospital-peer association networks are favorable factors for pursuing short-term and medium-term BF.

### Strengths and limitations

At the end of the present survey, we achieved our objectives of identifying the teaching and learning environment strategies proposed by midwives, seeking the perception of usefulness and skills development of breastfeeding women, assessing their influence on the feelings of selfesteem and commitment of breastfeeding women, and measuring the perception of usefulness and development of pedagogical skills of midwives compared to the educational interventions carried out. The qualitative data are very rich and open the idea of exploiting these data according to concepts of educational science. However, this study is also limited as follows. It is an exploratory study in which midwives and breastfeeding women from two maternity units were interviewed. Therefore, the level of usefulness, self-efficacy, self-esteem, and commitment to continued breastfeeding do not reflect the whole of France. We were not able to interview a larger number of hospital midwives. This was because access to hospitals was very limited during the health crisis. Our study could have been enriched by other issues raised by these midwives by interviewing the liberal midwives. Despite several follow-ups, some women did not respond to our one-month survey, which limited our ability to use the tests to compare in our quantitative analysis and forced us to group the different levels.

## CONCLUSIONS

The substantial discrepancies in women’s perceptions of the usefulness, self-efficacy, self-esteem, and commitment to breastfeeding between three days and 30 days postpartum illustrate the efficacy of the educational interventions conducted by midwives. To triangulate the results of the present study with our scoping review^25^, we will model an educational intervention to promote breastfeeding centered on women’s needs. There is an opportunity to design and implement an educational intervention based on pedagogical engineering regarding theoretical frameworks in pedagogy. Its evaluation, which should call for critical realism^35,36^, would then provide more specific information to help understand how an educational program based on pedagogical engineering can contribute, in the framework of health promotion, to promoting BF and achieving WHO 2030 goals.

## Data Availability

The data supporting this research are available from the authors on reasonable request.
